# Deletion of the CTRL2 Insulator in HSV-1 Results in the Decreased Expression of Genes Involved in Axonal Transport and Attenuates Reactivation *In Vivo*

**DOI:** 10.3390/v14050909

**Published:** 2022-04-27

**Authors:** Pankaj Singh, Matthew F. Collins, Richard N. Johns, Kayley A. Manuel, Ziyun A. Ye, David C. Bloom, Donna M. Neumann

**Affiliations:** 1 Department of Ophthalmology and Visual Sciences, University of Wisconsin-Madison, Madison, WI 53716, USA; psingh58@wisc.edu (P.S.); mfcollins@wisc.edu (M.F.C.); kmanuel@wisc.edu (K.A.M.); ye82@wisc.edu (Z.A.Y.); 2 Department of Molecular Genetics and Microbiology, University of Florida, Gainesville, FL 32611, USA; the_mountain667@yahoo.com (R.N.J.); dbloom@ufl.edu (D.C.B.)

**Keywords:** insulator, CTCF, rabbit ocular model, HSV-1 latency, epigenetics, chromatin

## Abstract

HSV-1 is a human pathogen that establishes a lifelong infection in the host. HSV-1 is transported by retrograde axonal transport to sensory neurons in the peripheral nervous system where latent viral genomes can reactivate. The resulting virus travels via anterograde axonal transport to the periphery and can cause clinical disease. CTCF insulators flank the LAT and IE regions of HSV-1 and during latency and maintain the integrity of transcriptional domains through a myriad of functions, including enhancer-blocking or barrier-insulator functions. Importantly, during reactivation, CTCF protein is evicted from the HSV-1 genome, especially from the CTRL2 insulator. CTRL2 is a functional insulator downstream of the 5′exon region of the LAT, so these results suggest that the disruption of this insulator may be required for efficient HSV-1 reactivation. To further explore this, we used a recombinant virus containing a deletion of the CTRL2 insulator (ΔCTRL2) in a rabbit ocular model of HSV-1 infection and induced reactivation. We show that, in the absence of the CTRL2 insulator, HSV-1 established an equivalent latent infection in rabbits, but those rabbits failed to efficiently reactivate from latency. Furthermore, we found a significant decrease in the expression of the gene Us9-, a gene that codes for a type II membrane protein that has been shown to be required for anterograde transport in neurons. Taken together, these results suggest that the functions of the CTRL2 insulator and Us9 activation in reactivating neurons are intrinsically linked through the regulation of a gene responsible for the axonal transport of HSV-1 to the periphery.

## 1. Introduction

Herpes Simplex Virus 1 (HSV-1) is a human pathogen that establishes a lifelong infection in the host. During the primary (acute) infection, the virus infects epithelial cells, where it replicates, produces progeny virus, and spreads. Following acute infection, HSV-1 is transported by retrograde axonal transport to sensory neurons in the peripheral nervous system, where it establishes a lifelong latent infection. Latent viral genomes can periodically reactivate in response to external and/or environmental stimuli, and following reactivation the virus travels via anterograde axonal transport to the periphery where it can cause clinical disease, including HSV-1-related ocular disease [1]. Importantly, repeated HSV-1 ocular episodes can ultimately lead to blindness [2].

During latency, HSV-1 genomes are chromatinized and associated with histones in sensory neurons [3,4,5,6]. The HSV-1 genome is essentially silenced except for the non-coding RNA known as the latency-associated transcript (LAT), which is expressed in a fraction of sensory neurons harboring latent genomes [7,8]. The LAT region of HSV-1 is populated with permissive euchromatin marks, including acetyl-H3K9K14 and H3K4me2 [5,6], while the nearby lytic regions, specifically the immediate early (IE) regions, bear repressive histone marks, such as H3K27me3 and H3K9me3, demarcating the transcriptionally repressed IE domains from the transcriptionally permissive regions of the latent genome [9,10,11,12,13]. However, in the early stages following the application of reactivation stimuli, the histone marks that are associated with the IE regions ICP0 and ICP4 rapidly undergo dynamic changes, and these genomic loci become more euchromatic in nature, likely allowing the expression of the IE genes and the subsequent reactivation from latency, indicating that the disruption of the latent transcriptional domains may be key to efficient reactivation [14,15,16]. Chromatin insulators, commonly known as CTCF insulators in mammalian cells, are involved in maintaining the transcriptional domains in latent HSV-1 genomes [17]. Initially described by Amelio, et al. in 2006, CTCF insulators flank the LAT and IE regions of HSV-1 and have since been characterized as enhancer-blocking and/or barrier insulators that are enriched in the insulator protein CTCF during latency [18,19,20,21]. Furthermore, the CTCF insulators flanking ICP0 and ICP4 differentially co-localize with the protein Suz12 from the PRC2 complex, likely to maintain the repressive chromatin environments of these gene regions during the latent infection in the sensory neurons [20]. Importantly, we recently showed that CTCF was differentially evicted from the sites flanking IE genes at very early stages following the application of reactivation stimuli in vivo, and [19] that the global depletion of CTCF using recombinant adeno-associated virus (rAAV) vectors resulted in the long-term persistent shedding of the virus in the absence of reactivation stimuli in rabbits, suggesting that the disruption of the CTCF insulators may be required for efficient HSV-1 reactivation through a mechanism yet to be defined [22].

To explore potential mechanistic roles of individual CTCF insulators in gene expression and reactivation, we generated a recombinant virus with the CTRL2 insulator deleted. Since this CTCF insulator is positioned downstream of the 5′exon region of the LAT, a genomic region containing the LAT promoter and an enhancer element, both of which are critical for reactivation [23], we hypothesized that the CTRL2 insulator was an important element that maintained latency in sensory ganglia. To support this hypothesis, we recently showed that the deletion of the CTRL2 insulator resulted in a decreased survival rate in mice, disrupted the efficient establishment of latency, and disrupted chromatin domain organization through altered repressive H3K27me3 histone mark distribution on the HSV-1 genome [24]. In these former studies, we used ChIP-*seq* on the latently infected trigemional ganglia (TG) of mice to show that there was a 2–4-fold *decrease* in the enrichment of the transcriptionally repressive histone marker H3K27me3 at the LAT and IE genes in the ΔCTRL2 recombinant virus, supporting that the CTRL2 insulator plays a fundamental role in maintaining the integrity of transcriptional domains of HSV-1 during latency [24]. Intriguingly, the ChIP-*seq* experiments in these previous studies also revealed that H3K27me3 enrichment *increased* in the recombinant virus in a region of the genome that was mapped near the inner tegument proteins UL36 and UL37, suggesting that the CTRL2 insulator was somehow interconnected with these genomic loci positioned more than 20K base pairs away [24]. It has been reported that cytosolic capsids associate with the inner tegument proteins UL36 and UL37 for their intracellular transport along microtubules to cytoplasmic membranes, where they meet other tegument and viral membrane proteins for secondary envelopment and virion formation. These proteins are essential for efficient capsid envelopment and fusion for the release of infectious particles and are involved in larger protein complexes that, together, are likely to be involved in axonal targeting for viral spread during reactivation [25,26]. Since H3K27me3 enrichment was not observed at UL36/37 in the wild-type (wt) virus 17Syn+, we further hypothesized that the CTRL2 insulator may also be involved in the virus’ ability to efficiently reactivate from latency, perhaps through the regulation of proteins involved in axonal transport. To further explore this possibility, we utilized the rabbit model of ocular HSV-1 infection and reactivation to determine: (1) whether the ΔCTRL2 recombinant virus displayed an attenuated reactivation phenotype in vivo; (2) the differences in the expression of genes involved in efficient axonal transport. Here, we showed that, in the absence of the CTRL2 insulator, HSV-1 established an equivalent latent infection compared to wt-infected rabbits, but those rabbits infected with the recombinant virus failed to efficiently reactivate from latency. Furthermore, while the expression of UL36 and UL37 were slightly decreased in the rabbits infected with the ΔCTRL2 recombinant relative to wt during reactivation, these differences were not statistically significant. On the other hand, there was a significant decrease in the expression of the gene Us9. This finding was exciting; HSV Us9 codes for a type II membrane protein that has been shown to be required for anterograde transport in neurons, and previous reports have shown Us9 mutants display a reduced axonal transport phenotype [27,28]. Taken together, these results suggest that the functions of the CTRL2 insulator (which is downstream of the reactivation-critical LAT enhancer element) and Us9 activation in reactivating neurons are intrinsically linked.

## 2. Material and Methods

### 2.1. Viruses and Cells

Vero cells (ATCC CCL-81) and Neuro 2A cells (ATCC CCL-131) were cultured in Dulbecco’s Modified Eagle’s Medium (DMEM) (Corning, NY, USA) supplemented with 10% fetal bovine serum (FBS) and 1% antibiotic-antimycotic solution (Gibco) in a humidified atmosphere with 5% CO_2_ at 37 °C and passaged at regular intervals when the cells were 75% confluent. HEK293FT cells (ThermoFisher R70007, Waltham, MA, USA) were cultured in DMEM supplemented with 10% FBS, 1% antibiotic-antimycotic solution and 2 mM L-glutamine in humidified atmosphere with 5% CO_2_ at 37 °C and passaged at regular intervals when the cells were 75% confluent. The HSV-1 strain, 17Syn+ (GenBank accession number NC_001806), was obtained from J. Stevens, propagated, and maintained in our lab. The recombinant HSV-1 (ΔCTRL2) was generated by the Bloom Lab and had a 135-bp deletion at the core CTRL2 insulator site in the wt 17syn+ genome, spanning nucleotides 120,500 to 120,635, as described in our previous publications [24,29]. HTS sequencing of the 17∆CTRL2 virus was performed, and the obtained data were compared to the sequence of our lab stock of 17Syn+. The results of this sequence analysis revealed no indels within the coding regions of genes, and only 1 single-nucleotide base change in UL36, which was synonymous. There were 4 additional single-nucleotide base substitutions and 1 two-nucleotide deletion that occurred in non-coding regions (mainly in reiterated regions in the repeats) [30]. Considering this sequencing data, it is unlikely that any sequence changes resulted in changes to the fitness of the ∆CTRL2 recombinant. All viruses were amplified and titrated on rabbit skin cells (CCL-68-ATCC) using Eagle’s Minimal Essential Medium (Life Technologies, Carlsbad, CA, USA) supplemented with 5% calf serum, 250 U of penicillin/mL, 250 μg of streptomycin/mL, and 292 μg of L-glutamine/mL (Life Technologies). 

### 2.2. Rabbit Ocular Infection Model

Male and female New Zealand White rabbits (2–3 kg) were infected in each cornea at doses of 200,000 PFU, as described previously [31]. Briefly, eyes were scarified and inoculated with 25 µL virus in MEM + 5% FBS and massaged for 30 s. Eyes were examined by split lamp microscopy (SLE) with 0.1% fluorescein on 3, 5, 7, 10, and 14 dpi, and assessed for the presence of ocular lesions. Lesion scores were determined by the surface area of the cornea for which the lesion occupied, as previously described [32]. Rabbits were considered latent when no corneal lesions were observed. Ocular swabs were performed to confirm that no infectious virus could be detected at 28 dpi. At 31 dpi, rabbits were subjected to trans-corneal iontophoresis of 0.01% epinephrine (0.8 mA for 8 min) daily, for three days, for reactivation purposes, as previously described [31,32]. Ocular swabs were taken from each eye daily for 12 days post-reactivation, and the presence of infectious virus was determined by plaque assay as a measure of reactivation. 

### 2.3. Determination of Pfu/Eye in Acutely Infected Rabbits

Ocular swabs to collect tear film from the rabbit eyes were performed daily from 1–7 dpi. Swabs were placed in collection tubes containing DMEM supplemented with 1% FBS and 1% antibiotic-antimycotic solution. The media was removed from the collection tubes and serially diluted 1:10 in DMEM with 1% FBS, then plated onto a confluent monolayer of vero cells in triplicate for 72 h for the quantitation of plaque-forming units. At 72 hpi the media were discarded, and cells were fixed in 10% buffered formalin and stained with crystal violet to visualize the plaque.

### 2.4. qPCR for Genome Copies/Ganglia

Rabbit ganglia were harvested at 28 dpi from wt or recombinant virus-infected animals, and total DNA was extracted using phenol-chloroform extraction techniques according to the manufacturer’s protocols. Genome copies per ganglia were measured by quantitative real-time PCR using primers and a probe specific to HSV-1 DNA polymerase. All real-time PCR experiments were performed using TaqMan Universal PCR Master Mix No AmpErase uracil *N*-glycosylase on an Agilent AriaMx Real-Time PCR using custom designed primer–probe mixes (Life Technologies) with the general protocol as follows: 95 °C for 10 min (1×) and then 95 °C for 15 s, followed by 60 °C for 1 min (45×). Threshold values used for PCR analyses were set within the linear range of PCR target amplification based on a standard curve generated for each plate. The relative HSV-1 copies for each sample were normalized to the relative copies of the host control rabbit GAPDH.

### 2.5. qRT-PCR Analysis

Rabbit trigeminal ganglia (TG) were isolated and placed in RNA*later* and stored according to the manufacturer’s specifications. RNA was extracted by removing RNA*later* from the samples and adding Trizol reagent (Sigma-Aldrich, St. Louis, MO, USA) to each sample. Briefly, each TG was homogenized in 1.2 mL Trizol and, following the addition of 0.2 volume of chloroform, samples were centrifuged for phase separation. RNA was precipitated from the aqueous phase using a volume of 0.7 isopropanol, followed by DNase treatment using DNA-*free* (Ambion, Austin, TX, USA), according to the manufacturer’s directions. One-step qRT-PCR was performed using the SuperScript III One-Step RT-PCR system with Platinum Taq DNA Polymerase or SuperScript III Sybr green, according to the manufacturer’s protocol (Life Technologies). Real-time PCR was performed on cDNA according to the above-described procedures and protocols using the primers and probes listed in Table 1. 

### 2.6. Prediction of Putative CTCF Insulators

Potential CTCF-binding sites were predicted by CTCFBSDB 2.0 [33], a collection of previously identified core binding motifs that are represented by position weight matrices (PWM). Fragments of the HSV-1 17Syn+ sequence were entered as input into the CTCFBS Prediction Tool, which uses PWM to report the sequence and orientation of the best hits. Only hits with a PWM score >3 were considered as potential CTCF-binding sites in this study, and were subsequently subjected to cloning [33]. 

### 2.7. Plasmid Constructs and Enhancer Blocking Assays

The HSV-1 LAT enhancer (nucleotides (nt) 118,888 to 119,477) was directionally cloned into the 5′ KpnI and 3′ SacI polylinker sites of the pGL3-simian virus 40 (SV40) promoter control vector (Promega) using PCR-generated KpnI and SacI linkers to generate the pGL3-control/LTE enhancer plasmid, as previously described [18]. This construct was then used as a positive control in all transient transfection assays. Additional test constructs were generated to test the enhancer-blocking abilities of the CTUS1 region of HSV-1. First, a 190 bp fragment (nt 143,691 to 143,880) was generated by PCR with NheI and XhoI linkers. The pGL3-control/CTUS1A and pGL3-control/LTE + CTUS1A plasmids were generated by directionally cloning the CTUS1A fragment into the 5′ NheI and 3′ XhoI sites of the pGL3-SV40 promoter control vector or the pGL3-control/LTE enhancer using PCR-generated NheI and XhoI linkers, respectively. Next, a 203 bp fragment (nt 143,870 to 144,072) containing the predicted CTCF-binding sites (nt 143,893 to 143,913 and 144,026 to 144,035) downstream of the core CTUS1 CTCF-binding cluster was generated by PCR with NheI and XhoI linkers. The pGL3-control/CTUS1B and pGL3-control/LTE + CTUS1B plasmids were generated by directionally cloning the CTUS1B into the 5′ NheI and 3′ XhoI sites using PCR-generated NheI and XhoI linkers to create the indicated plasmids. Transient transfections were set up with 500 ng of each luciferase test construct described above in 24-well dishes seeded 48 h earlier with HEK293FT cells (R70007; ThermoFisher. Massachusetts, USA) and Neuro-2a cells (CCL-131; ATCC) at 9 × 10^4^ cells/well. Then, 500 ng of pMCS-Green *Renilla* Luc vector (ThermoFisher) was co-transfected as a control for transfection efficiency. Each luciferase construct was tested in technical triplicate and repeated 3 times. Transfections were carried out using 0.75 μL of Lipofectamine 3000 Reagent and 1 μL of P3000 Reagent (Invitrogen, Waltham, MA, USA) per reaction well, following the manufacturer’s protocols. Following a 48 h incubation, the cells were lysed and luciferase levels were tested using a Dual Luciferase Reporter Assay System (Promega) following the manufacturer’s protocols, and the luciferase levels were measured using a Tecan SPARK Microplate Reader using SparkControl Software.

### 2.8. Ethics Statement

Protocols for the rabbit ocular infection experiments were approved by the University of Wisconsin-Madison Institutional Animal Care and Use Committee. New Zealand White rabbits (1.5–2.5 kg) were used in all experiments and were handled and maintained in accordance with the tenets established by the Association for Research in Vision and Ophthalmology in its resolution on the care and use of animals in research.

### 2.9. Statistics Methods

All statistics were performed using GraphPad/Prism (version 8.3.0). The significance of the experiments with one time point was determined by unpaired *t*-tests where noted (* *p* < 0.05, ** *p* < 0.01, *** *p* < 0.001, **** *p* < 0.0001, ns = not significant). Significance for all rabbit experiments, including gene expression and qPCR, were determined by ordinary one-way ANOVAs. An exact version of chi-square analysis was performed for the analyses of reactivation data obtained for all pairwise comparisons with the control (in this case, wt-infected animals).

## 3. Results

**Figure 1:**
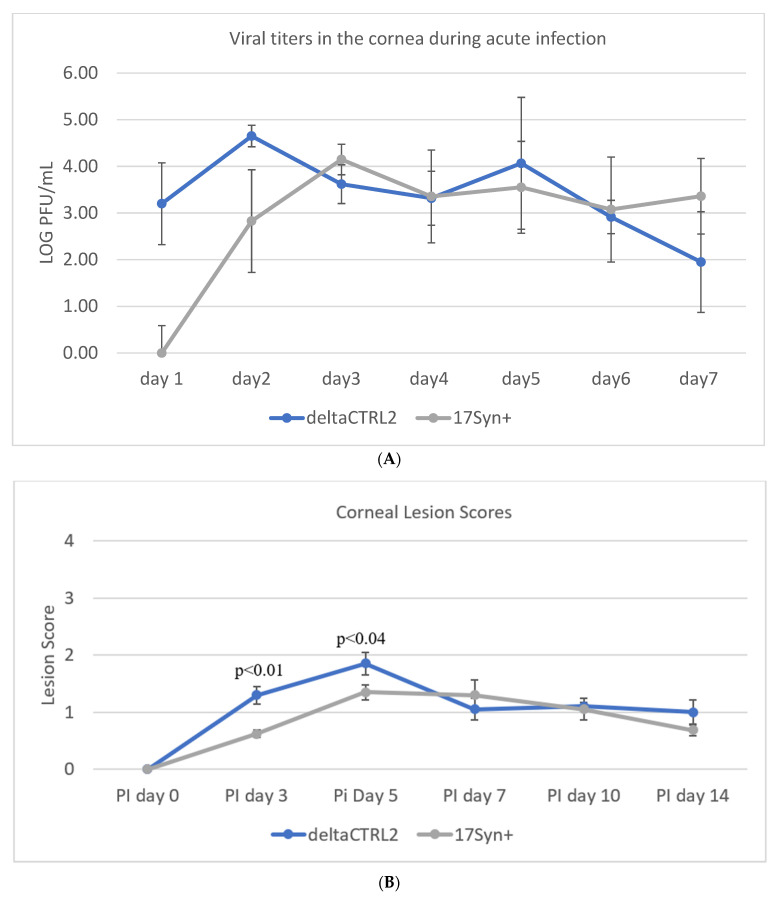
Ocular replication of wild-type 17Syn+ and ΔCTRL2 in rabbit corneas. (**A**) Ocular swabs were taken following infection from 1–7 dpi and plaque-forming units were calculated for both cohorts of rabbits during the acute infection. There was a significant increase in pfu/eye from the swabs of the ΔCTRL2-infected rabbits on 1–2 dpi. (**B**) Slit lamp examinations were performed on a separate cohort of rabbits to assess the HSV-1 lesion size and severity between the two virally infected groups. Similar to the pfu/eye, there were higher scores for rabbits infected with ΔCTRL2 on days 3–5 post-infection, indicating the lesions in those eyes were more significant in the earlier days. Statistical analyses were performed by one-way ANOVA (*n* = 10).

**Deletion of the CTRL2 Insulator Does Not Significantly Alter Viral Replication in the Cornea During the Acute Infection in Rabbits.** Previously, we showed that the ΔCTRL2 recombinant virus displayed a replication defect in rabbit skin cells and other epithelial cells in in vitro experiments at lower MOI [24]. To determine whether this defect in replication could also be observed in vivo in rabbit corneas, we infected New Zealand White rabbits with either the wt 17Syn+ or the ΔCTRL2 recombinant virus at 200,000 pfu/eye. Rabbits were sedated and both corneas were anesthetized. Viral stocks were applied following corneal abrasion. After 24 hpi, the rabbit eyes were swabbed and the tear film was plated on vero cells using 1:10 serial dilutions in order to quantitate the number of pfu/eye from 1–7 dpi acute infection. Plaque assays showed that, on 1 and 2 dpi, there were significantly higher pfus in the swabs obtained from rabbits infected with the ΔCTRL2 recombinant compared to the wt virus, but by 3 dpi both the wt and mutant viruses had equivalent pfu/eye (Figure 1A). In a separate cohort of rabbits, the presence and severity of ocular lesions were also evaluated by slit lamp microscopy for the comparison of wt and ΔCTRL2 recombinant viral infections of the cornea. Ocular lesion scores were determined using methods and scoring criteria previously described [32,34,35]. Consistent with the infectious virus quantification from 1–7 dpi, the average slit lamp scores were significantly higher initially for the eyes of rabbits infected with the ΔCTRL2 recombinant virus compared to the wt, but by 5 dpi there was no longer a significant difference in the slit lamp scores, suggesting that the initial burst of viral replication in the cornea of rabbits infected with the ΔCTRL2 recombinant is short-lived, and does not induce a more virulent phenotype in the cornea over time (Figure 1B). Taken together, these data suggest that viral fitness and viral replication in vivo, at least in the cornea of rabbits, are not significantly altered by the deletion of the CTRL2 insulator.

**Figure 2:**
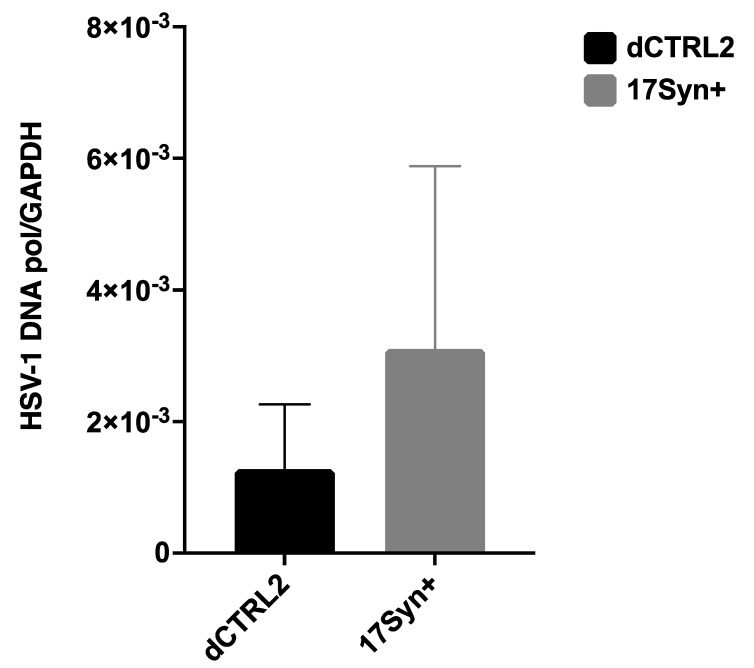
HSV-1 genome copies in rabbit ganglia in wt 17Syn+ and the ΔCTRL2 recombinant following infection. The number of HSV-1 genomes per ganglia was determined between wt 17Syn+ and ΔCTRL2 viral infections in rabbits. Relative copies of HSV-1 DNA polymerase were determined by PCR at 28 dpi. qPCR data is presented as a ratio of HSV-1 DNA pol/rabbit GAPDH. One-way ANOVA of independent samples was used to determine the statistical significance between the mutant and wt (*n* = 6).

**There Are No Statistically Significant Differences in HSV-1 Genome Loads in the Sensory Neurons of the Trigeminal Ganglia (TG) During Latency in the** Δ**CTRL2 Recombinant Compared to wt.** In a previous report, we showed that the deletion of the CTRL2 insulator contributed to a significant decrease in the survival of mice, suggesting that latency was not established as efficiently in ΔCTRL2-infected mice [24]. We also quantitated significantly lower viral genomes per ganglia in the mice infected with the ΔCTRL2 recombinant compared to the wt at 28 dpi, suggesting that the CTRL2 insulator was required for the efficient establishment of latency in vivo in the ocular mouse model of HSV-1 infection. To determine if these findings were consistent between animal models, we also harvested the TG from rabbits infected with either the ΔCTRL2 recombinant or wt virus at 28 dpi and isolated DNA. qPCR was performed using the HSV-1 DNA polymerase gene (UL30) to quantify viral genomes per ganglia (Table 2). All values were normalized to the host control, rabbit GAPDH. In contrast to our previous findings in relation to the TG of mice, there were ~2-fold fewer HSV-1 genomes in ganglia harvested from rabbits infected with the ΔCTRL2 recombinant, but this decrease was not statistically significant by analysis of variance statistical means (Figure 2).

**Figure 3:**
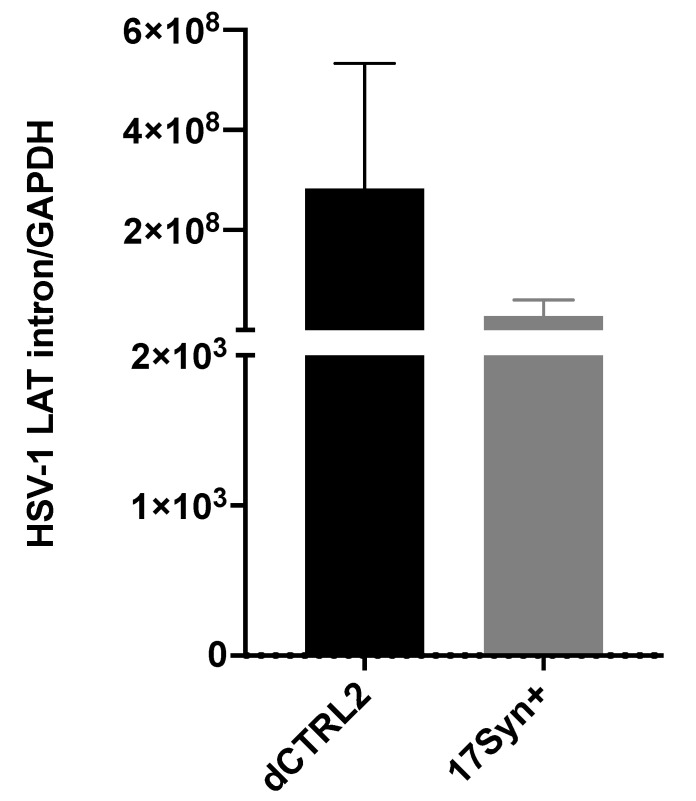
LAT intron expression was significantly higher in the ΔCTRL2-infected rabbits during latency. Rabbit TG were isolated and RNA extracted, as indicated in the methods section, and subsequently used for qRT-PCR to assess gene expression during latency for the wt and recombinant viruses. Relative values for LAT intron were normalized to rabbit GAPDH and are presented as normalized ratios for gene expression. One-way ANOVA of independent samples was used to determine statistical significance between the mutant and wt (*n* = 6). Data presented here are consistent with previous reports in the mouse model [24]. There was a significant increase in LAT intron expression in the ΔCTRL2-infected rabbits compared to wt-infected animals at latency.

**Figure 4:**
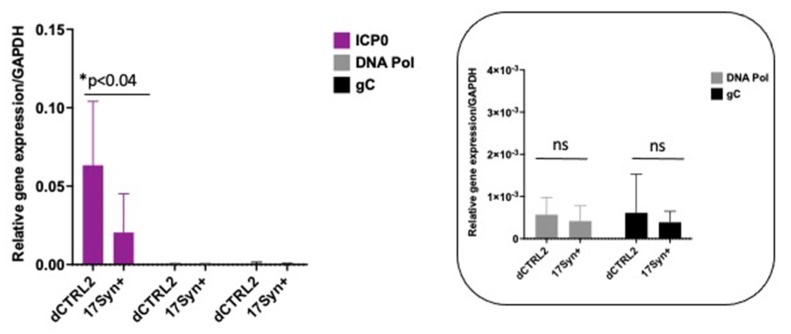
ICP0 expression was significantly higher in the ΔCTRL2-infected rabbits during latency, but early and late gene expression was consistent between the two virally infected cohorts. Rabbit TG were isolated and RNA was extracted as indicated in the methods section, and subsequently used for qRT-PCR to assess gene expression during latency for the wt and recombinant viruses. Relative values for ICP0, DNA pol and gC were normalized to rabbit GAPDH and are presented as normalized ratios for gene expression (**inset**). ICP0 expression was significantly higher than DNA pol and gC. The y-axis scale was adjusted to account for the increase in ICP0 expression. One-way ANOVA of independent samples was used to determine the statistical significance between the mutant and wt samples (*n* = 6). Data presented here are consistent with previous reports of the mouse model. There was a significant increase in ICP0 expression in the ΔCTRL2-infected rabbits compared to wt-infected animals at latency. * indicates a significant reduction in luciferase expression between the LTE and LTE/CTCF construct.

**LAT intron and ICP0 expression are significantly higher in rabbits latently infected with the** Δ**CTRL2 recombinant**. In our previous studies using the TG of mice, we found significantly increased expressions of the ICP0 and ICP27 genes (*p* < 0.05). Moreover, the expression of the LAT intron at 28 dpi was consistent with the previously reported findings of Lee et al. using a CTRL2 recombinant virus from the KOS parent background [21]. Because we observed what appeared to be host-related differences with respect to genome copies between the rabbit and mouse models of HSV infection, we also quantitated viral gene expression in latently infected rabbit ganglia to determine if the ΔCTRL2 recombinant infections in rabbits produced a similar gene expression phenotype that was observed in the latent ganglia of mice. Rabbit ganglia were harvested at 28 dpi and RNA was isolated and subjected to qRT-PCR using primer/probe sets specific for either the LAT intron, ICP0, HSV-1 DNA pol or gC (Table 1) to represent all kinetic classes of genes in the HSV-1 genome. Consistent with our mouse data, we found significant increases in both LAT intron and ICP0 expression in the rabbit ganglia infected with the ΔCTRL2 recombinant compared to wt. The expression of the LAT intron was abundant for both the wt and recombinant viruses, consistent with the establishment of latency, yet we observed a significant ~10-fold increase in LAT intron expression in the ΔCTRL2 recombinant compared to wt expression (Figure 3). The expression of the IE gene ICP0 was markedly lower than that of LAT intron, as expected since the rabbit ganglia were harvested prior to the application of reactivation stimuli. Nevertheless, the ICP0 expression was significantly higher (~3.5-fold) in the ΔCTRL2 recombinant compared to wt (Figure 4). In contrast, we found no significant differences in either E or L gene expression during the latent infection in rabbits, indicating that the changes observed in gene expression by the ΔCTRL2 recombinant were limited to the LAT and IE genes (Figure 4). These findings are consistent with our previous findings in mice, where significantly elevated LAT intron and ICP0 gene expressions were measured for the ΔCTRL2 recombinant and correlated with the reduced H3K27me3 enrichment of the ΔCTRL2 recombinant viral genome in mice, suggesting that a similar chromatin profile is present in rabbit ganglia infected with the ΔCTRL2 recombinant.

**Reactivation was significantly attenuated in rabbits latently infected with the** Δ**CTRL2 recombinant**. The goal of the current study was to determine whether the deletion of the CTRL2 insulator in HSV-1 resulted in a phenotypic change in the reactivation of rabbits infected with the deletion virus. The advantage of the rabbit model for quantifying efficient HSV-1 reactivation was that the latent virus could be efficiently and synchronously induced to reactivate the following transcorneal iontophoresis of ß-adrenergic agonists, such as epinephrine [31,36]. Latently infected rabbits infected with either the wt or the ΔCTRL2 recombinant following ocular scarification were subjected to the transcorneal iontophoresis of epinephrine (TCIE), starting on 31 dpi for three consecutive days. Rabbit eyes were swabbed daily for 12 days to collect tear film and these swabs were subjected to a round of freeze-thaws and then assayed for the presence of infectious virus to measure reactivation. Exact chi-square analyses for all pairwise comparisons with the control (in this case, wt-infected animals) were performed, showing significant decreases in the number of swabs, eyes and rabbits that shed the infectious virus in the ΔCTRL2 recombinant group (Table 2). These data confirm that reactivation was attenuated by the deletion of the CTRL2 insulator, and further suggest that the CTRL2 insulator is an important element contributing to efficient reactivation from latency.

**Figure 5:**
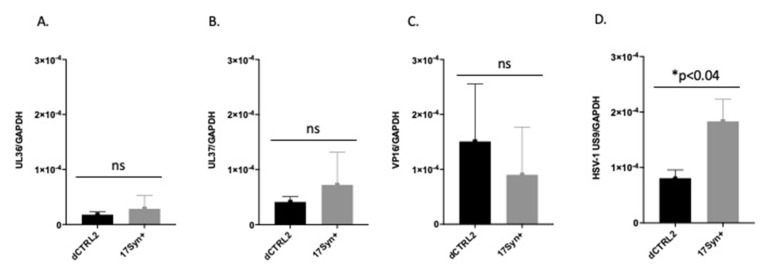
Us9 expression was significantly lower in ΔCTRL2-infected rabbits at 5 days post-reactivation. Rabbit TG were isolated and RNA extracted as indicated in the methods section, and subsequently used for qRT-PCR to assess gene expression during latency for the wt and recombinant viruses. (**A**) Relative values for UL36 were normalized to rabbit GAPDH and are presented as normalized ratios for gene expression. (**B**) Relative values for UL37 were normalized to rabbit GAPDH and are presented as normalized ratios for gene expression. (**C**) Relative values for VP16 were normalized to rabbit GAPDH and are presented as normalized ratios for gene expression. (**D**) Relative values for Us9 were normalized to rabbit GAPDH and are presented as normalized ratios for gene expression. One-way ANOVA of independent samples was used to determine statistical significance between the mutant and wt (*n* = 5). * indicates a significant reduction in luciferase expression between the LTE and LTE/CTCF construct.

Us9 expression was significantly reduced following the application of the reactivation stimuli in rabbits latently infected with the ΔCTRL2 recombinant. Previous ChIP-*seq* experiments revealed that H3K27me3 enrichment *increased* in the TG of mice infected with the ΔCTRL2 recombinant in a region of the genome that was mapped near the inner tegument proteins UL36 and UL37, suggesting that the CTRL2 insulator was somehow interconnected with these genomic regions. It has been proposed by others that the UL36 and UL37 tegument proteins are involved in and integral to axonal targeting for viral spread during reactivation [25,26]. Therefore, we hypothesized that we would observe a decrease in the expression of either UL36 and/or UL37 in ganglia harvested from rabbits latently infected with ΔCTRL2 in the early stages of reactivation, compared to wt-infected animals. To quantify this, we applied TCIE to rabbits infected with the mutant or wt viruses and then harvested TG at 5 days post-reactivation, since this was the time where we could consistently detect ocular swabs that were positive for the presence of infectious virus in wt-infected animals. RNA was isolated and subjected to qRT-PCR using primers for either UL36 or UL37. Surprisingly, while there was a slight reduction in the levels of UL36 and 37 transcripts in the ganglia of ΔCTRL2-infected animals compared to wt, these reductions were not statistically significant (Figure 5A,B). In addition, we measured the expression of VP16 in these reactivated ganglia, since VP16 is required for efficient reactivation from latency. Again, we found no significant differences in VP16 expression between the wt and mutant-infected ganglia (Figure 5C). However, in re-evaluating the literature for elements required for the efficient anterograde transport of HSV-1, we found key reports by the Johnson lab that showed that the type II membrane protein Us9 plays a fundamental role in efficient anterograde axonal transport, suggesting its importance in the process of reactivation from latency [28]. Therefore, we quantitated Us9 expression in the ganglia harvested from rabbits subjected to reactivation stimuli at 5 days post-reactivation. We found a striking and significant decrease in Us9 expression in rabbit ganglia infected with ΔCTRL2 at 5 days post-reactivation relative to wt-infected and reactivated ganglia, consistent with the attenuated reactivation phenotype observed for the ΔCTRL2-infected animals (Figure 5D). These data further suggest that the function of the CTRL2 insulator near the LAT enhancer may be intrinsically linked to the ability of viral genomes to reactivate from latency.

**Figure 6:**
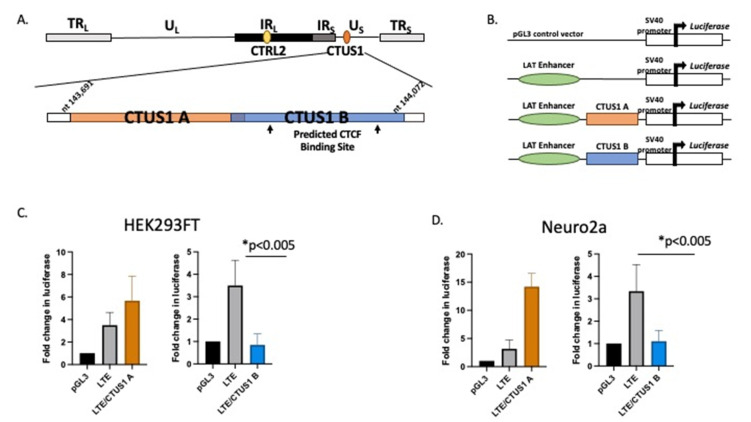
A putative CTCF insulator was identified in the US region of HSV-1 and characterized as an enhancer blocker. Luciferase reporter constructs were generated to test the ability of the CTCF-binding motif to block the LAT enhancer. (**A**) The CTCFBDBS 2.0 program was used to identify the CTUS1B site as a putative CTCF-binding site. The CTUS1A site corresponds to a ~200 bp sequence not predicted to have insulator function (negative control for enhancer-blocking activity). Each CTCF/LTE construct contained ~200 bp of HSV-1 Us region-specific sequences positioned between the LAT enhancer element and the SV40 promoter in thAe pGL3 control vector. (**B**) Reporter constructs were generated using the commercially available pGL3-control vector (Promega). Constructs contained ~200 bp sequences of the CTUS1A or CTUS1B fragments identified in A, in the presence of the enhancer element in the 5′exon region of LAT (LTE). (**C**) Reporter assays in HEK293FT cells. All transfections were completed in triplicate wells and were repeated 5 times (*n* = 3). Data are presented as both a comparison of the LTE to the LTE/CTCF construct and the CTCF compared to the LTE/CTCF construct. * indicates a significant reduction in luciferase expression between the LTE and LTE/CTCF construct; *p* values <0.001, determined by unpaired one-tailed Student’s *t*-tests in pairwise comparisons. All luciferase values were normalized to the pGL3 control, which was set to 1. (**D**) Reporter assays were performed in neuro 2a cells. All transfections were completed in triplicate wells and were repeated 5 times (*n* = 3). Data are presented as both a comparison of the LTE to the LTE/CTCF construct and the CTCF compared to the LTE/CTCF construct; * indicates a significant reduction in luciferase expression between the LTE and the LTE/CTCF construct where *p* < 0.001, determined by unpaired one-tailed Student’s *t*-tests in pairwise comparisons.

**A 200 bp fragment in the Us Region of HSV-1 (upstream of Us9) had LAT-enhancer blocking activity.** The decreased expression of Us9 in rabbits infected with the ΔCTRL2 recombinant suggested that the CTRL2 insulator was involved in regulating Us9 transcription either during latency and/or reactivation. CTCF insulators that have been characterized in the context of mammalian cells and transcriptional control are known to self-dimerize to form complex three-dimensional chromatin structures known as *chromatin loops* that orient distance-separated transcriptional elements such as enhancers and promoters into close spatial proximity for the purposes of transcriptional control [37,38]. Given that the CTRL2 insulator and Us9 are also distance separated in the HSV-1 genome, we hypothesized that there might be a second functional insulator element near the Us9 region that could potentially facilitate CTCF dimerization during latency. In fact, a putative CTCF-binding motif was previously described in the Us region of the genome [18]. To further explore this possibility, we utilized an online CTCF insulator prediction database known as CTCFBSDB 2.0 to identify putative CTCF insulators in the HSV-1 Us region near the Us9 gene. In the HSV-1 sequence spanning nucleotides 143,691–144,072, we identified a putative CTCF insulator from the database (Figure 6A-CTUS1B). To test the ability of the predicted CTCF insulator to block LAT enhancer activity, we generated reporter constructs using a commercially available pGL3-control plasmid and tested each construct for enhancer-blocking function against the LAT enhancer using standard luciferase assays in transient transfections in either HEK293 or Neuro 2A cells to account for cell-type-specific activity between the epithelial and neuronal cells (Figure 6B). The reporter constructs tested included the putative CTCF motif with ~200 bp of site-specific sequences flanking the core insulator motif cloned into a construct containing a 589 bp sequence that corresponded to the LAT enhancer of HSV-1 (LTE), as previously reported [18,19,20]. An additional construct, containing a 200 bp sequence from the Us region of HSV-1 that was not predicted to be a CTCF insulator by the CTCFBSDB 2.0 database, was also generated and served as a control plasmid (Figure 6-CTUS1A). Here, we found that, in both HEK293 and Neuro 2A cells, the CTUS1B construct was able to significantly decrease luciferase expression compared to the LAT enhancer (LTE), indicating that this site was an enhancer-blocking insulator (Figure 6C,D). These data suggest that there is a functional insulator near the Us9 region of the genome that could be important for regulating region-specific gene expression during latency.

## 4. Discussion

CTCF insulators are important regulatory domains that have extensive roles in the transcriptional control of eukaryotic gene expression. The role of chromatin insulators in the context of DNA viruses has become an intense area of focus recently; investigators have shown that these insulators establish chromatin barriers, recruit co-activating or co-repressing protein complexes, and can self-dimerize to form chromatin loops that regulate transcription over large genomic spans [3,39,40,41,42,43]. To this end, we have previously shown that there are multiple putative CTCF insulators in the HSV-1 genome, that the protein CTCF is differentially bound to these sites, and that many have enhancer-blocking function, suggesting that they are functional insulators that are involved in the transcriptional control of HSV-1 during latency [18,19,20,24]. Importantly, we have also reported that, during the early stages post-reactivation in mice, CTCF is evicted from the insulator sites in the HSV-1 genome [19], and that the depletion of CTCF in neurons of latently infected rabbits results in the long-term shedding of the infectious virus at the periphery and an increase in ICP0 expression [22], suggesting that CTCF eviction is an important step in reactivation.

**Figure 7:**
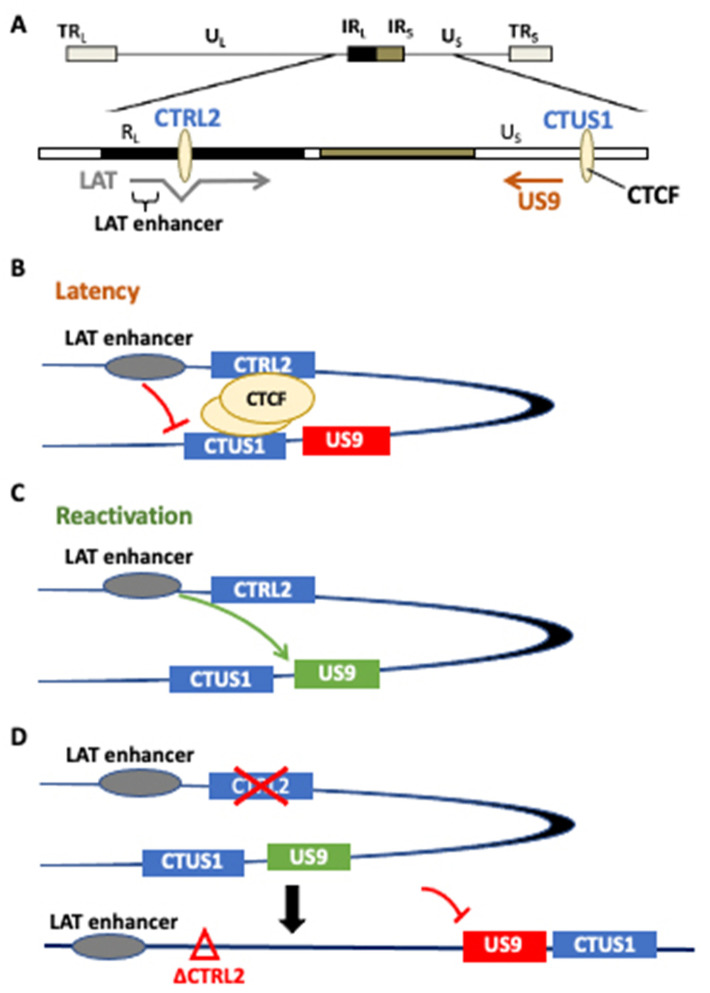
(**A**) A potential model for the CTRL2-regulated gene expression of Us9. We hypothesize that the CTRL2 insulator dimerizes with CTUS1 near the Us9 region of HSV-1 to establish a chromatin loop that would support positioning the LAT enhancer in close proximity to Us9. Our model proposes the following mechanism for HSV-1 reactivation from neurons. (**B**) During latency, CTCF insulators CTRL2 and CTUS1 self-dimerize to position the LAT enhancer in spatial proximity to the Us9 gene. During latency, these insulators are occupied by CTCF protein, acting both as an enhancer blocker to the LAT enhancer and preventing the expression of Us9. (**C**) In reactivation, the CTCF protein is evicted, thereby allowing for the LAT enhancer to activate the Us9 promoter. (**D**) In the absence of the CTRL2 insulator, the spatial orientation between the LAT and Us9 regions is disrupted, inhibiting efficient reactivation through a mechanism involving axonal transport.

To further explore the roles of the individual CTCF insulators in HSV-1 transcriptional control, we generated a recombinant virus with the CTRL2 insulator deleted from the 17Syn+ parent virus, a well-characterized parent strain of HSV-1 known to be a virulent and reactivating strain of HSV-1 in both mice and rabbit ocular models of infection. This recombinant was extensively phenotyped in vitro and in vivo in the mouse model during the acute and latent stages of infection. Following ChIP-*seq* experiments in latently infected mice, we showed that H3K27me3 enrichment was attenuated in the LAT and IE gene regions, consistent with the increase in gene expression observed in the latent TG of mice, but that H3K27me3 enrichment *increased* at the genomic loci corresponding to the UL36 and UL37 genes, suggesting that the CTRL2 insulator might be involved in the expression of these two inner tegument genes [24]. Importantly, UL36 and UL37 are essential for efficient capsid envelopment and fusion for the release of infectious particles and are involved in larger protein complexes, and are together involved in the axonal targeting of viral spread during reactivation. We hypothesized that the CTRL2 insulator may also be involved in efficient reactivation from latency through the regulation of genes involved in axonal transport. While there was a slight decrease in UL36 and UL37 expression in ΔCTRL2-infected rabbits, these decreases were not significant, thus suggesting that there may be other co-factors involved in reducing reactivation in the absence of the CTRL2 insulator. To further support this, we quantitated a significant decrease in the expression of Us9 in rabbits infected with the mutant virus. This finding was exciting. >HSV Us9 is a type II membrane protein that has been shown to be required for anterograde transport in neurons, and previous reports have shown that Us9 mutants display a reduced axonal transport phenotype [27,28]. Consistent with these previous reports, in the current study, we showed that rabbits infected with the CTRL2 deletion virus had a significantly attenuated reactivation phenotype compared to the wt-infected animals. It is important to point out that, in the current study, we measured clinical reactivation as quantitated by the presence of infectious virus recovered in the eyes of rabbits following the induction of reactivation. More extensive studies that quantitate the initiation of reactivation or molecular reactivation in the neuron are currently underway, and will be the subject of an additional manuscript in the future. Nonetheless, the presented data suggest that the CTRL2 insulator plays a role in suppressing Us9 expression during latency, and perhaps allows Us9 expression during reactivation episodes in neurons. Considering that the Us9 region of the genome is distance separated from the CTRL2 insulator, it is intriguing to speculate that these two regions may be brought together into close spatial proximity by the dimerization of CTCF insulators. CTCF insulators also establish and maintain higher-order chromatin structures, known as chromatin loops [44]. In eukaryotic cells, genomes are folded into spatial domains in three-dimensional forms and organized into higher-order chromatin structures that are subdivided epigenomic compartments ranging in size from 6 kb to more than 5 Mb. These compartments are known as topologically associated domains (TADs) and function as interaction hubs where regulatory elements interact with genes to activate or silence transcription [45,46]. TADs are partitioned into transcriptional domains by the self-dimerization of complementary CTCF insulators [44], and more recent evidence has shown that these DNA loops are anchored by protein complexes known as cohesin [44]. We have recently shown that a cohesin protein is enriched near the CTRL2 insulator, further supporting that this insulator can form a chromatin loop if in the right spatial orientation to self-dimerize with additional CTCF insulators [29]. We hypothesized that this insulator might be interacting with another insulator near the Us9 region of HSV-1 to establish a chromatin organization that would support the positioning the LAT enhancer in close proximity to Us9. To support this, we identified a CTCF-binding site in the Us region of HSV-1 that had enhancer-blocking ability in a cell-type-independent manner that was occupied by CTCF during latency [18]. Therefore, we propose the following model as a potential mechanism for HSV-1 reactivation from neurons (Figure 7). During latency, CTCF insulators CTRL2 and CTUS1 self-dimerize to position the LAT enhancer in spatial proximity to the Us9 gene. During latency, these insulators are occupied by the CTCF protein, acting both as an enhancer blocker to the LAT enhancer and preventing the expression of Us9 (Figure 7B) [18,19]. However, upon reactivation, CTCF protein is evicted, thereby allowing for the LAT enhancer to activate the Us9 promoter (Figure 7C). In the absence of the CTRL2 insulator, the spatial orientation between the LAT and Us9 regions is disrupted, inhibiting efficient reactivation through a mechanism involving axonal transport (Figure 7D). Further studies that will map these spatial interactions in vivo are currently underway.

## 5. Conclusions

The deletion of the CTRL2 insulator resulted in attenuated reactivation from latency in the rabbit ocular model of HSV-1. We identified a CTCF insulator-like element near the Us9 region of HSV-1, a gene region important for efficient anterograde axonal transport and reactivation from neurons. This element was capable of an enhancer-blocking function, suggesting that it is a functional insulator element. We showed that, in the absence of the CTRL2 insulator, Us9 expression was significantly lower, a finding consistent with attenuated reactivation. These findings support the possibility that the LAT enhancer element may be brought into close spatial proximity to the Us9 gene for transcriptional control.

## Figures and Tables

**Table 1:** List of primer sequences.

Gene Name	Primer Sequence 5′–3′
LAT intron	FP: 5′-ACC CAC GTA CTC CAA GAAGGC RP: 5′-TAA GAC CCA AGC ATA GAG AGC CA-3′ Probe: 5′-/56-FAM/TCC CAC CCC GCC TGT GTT TTT/3BHQ1/-3′
HSV-1 DNA Pol	FP: 5′-AGA GGG ACA TCC AGG ACT TTG T-3′ RP: 5′-CAG GCG CTT GTT GGT GTA C-3′ Probe: 5′-/56-FAM/ACC GCC GAA CTG AGC A/3BHQ/-3′
gC	FP: 5′-CCT TGC CGT GGT CCT GTG GA-3′ RP: 5′-GGT GGT GTT GTT CTT GGG TTT G-3′ Probe: 5′-/56-FAM/CCC CAC GTC/ZEN/CAC CCC CGA CC/3IABKFQ-3′
ICP0	FP: 5′-AGC GAG TAC CCG CCG GCC TG-3′ RP: 5′-CAG GTC TCG GTC GCA GGG AAA C-3′ Probe: 5′-/56-FAM/AGC CCG CCC CGG ATG TCT GGG/3BHQ 1/-3′
VP16	FP: 5′-CCT CGA TGG TAG ACC CGT AA-3′ RP: 5′-ACA TTC GCG AGC ACC TTA AC-3′ Probe: 5′-/56-FAM/CAT AAA GTA CCC AGA GGC/3BHQ 1/-3′
UL36	FP: 5′-CTG CTG TTG TAG GCG GTA AC-3′ RP: 5′-ACC AAC GAA CCA TTC AGT GC-3′
UL37	FP: 5′-CAC TGC ACG CAC TAC CTT TC-3′ RP: 5′-ATA GTA CCC GTA TTC CCG CG-3′
US9	FP: 5′-CCA ACA GTC GGT ATT AAG GCG-3′ RP: 5′-AGA GAC GAC AAG AAG GAC GC-3′

**Table 2:** *In vivo* reactivation following the transcorneal iontophoresis of epinephrine ^&^.

Virus	No. Rabbits Positive/Total (%)	No. Eyes Positive/Total (%)	No. Swabs Positive/Total (%)
ΔCTRL2	2/7 (28.6%) *	3/14 (21.4%) *	6/126 (4.8%) *
17Syn+	6/6 (100%)	8/12 (66.7%)	27/108 (25%)

^&^ Rabbits were subjected to transcorneal iontophoresis of epinephrine for 3 consecutive days to reactivate the virus. Eyes were swabbed daily, and positive swabs reflect that virus was detected in the plaque assays on vero cells. The total number of swabs indicates the cumulative number of swabs that were performed over 12 days, with positive swabs reflected in the numerator. * An exact version of chi-square analysis was performed for all pairwise comparisons with the control (in this case, wt-infected animals); *p* < 0.005.

## Data Availability

Not applicable.
